# Modified oxylipins as inhibitors of biofilm formation in *Staphylococcus epidermidis*

**DOI:** 10.3389/fphar.2024.1379643

**Published:** 2024-05-23

**Authors:** Jacquelyn E. Peran, Lilibeth A. Salvador-Reyes

**Affiliations:** Marine Science Institute, College of Science, University of the Philippines Diliman, Quezon City, Philippines

**Keywords:** oxylipins, fatty acids, biofilm, *Staphylococcus* *epidermidis*, antimicrobial

## Abstract

New approaches to combating microbial drug resistance are being sought, with the discovery of biofilm inhibitors considered as alternative arsenal for treating infections. Natural products have been at the forefront of antimicrobial discovery and serve as inspiration for the design of new antibiotics. We probed the potency, selectivity, and mechanism of anti-biofilm activity of modified oxylipins inspired by the marine natural product turneroic acid. Structure-activity relationship (SAR) evaluation revealed the importance of the *trans-*epoxide moiety, regardless of the position, for inhibiting biofilm formation. *trans*-12,13-epoxyoctadecanoic acid (**1**) and *trans*-9,10 epoxyoctadecanoic acid (**4**) selectively target the early stage of biofilm formation, with no effect on planktonic cells. These compounds interrupt the formation of a protective polysaccharide barrier by significantly upregulating the *ica* operon’s transcriptional repressor. This was corroborated by docking experiment with SarA and scanning electron micrographs showing reduced biofilm aggregates and the absence of thread-like structures of extrapolymeric substances. *In silico* evaluation revealed that **1** and **4** can interfere with the AgrA-mediated communication language in Staphylococci, typical to the diffusible signal factor (DSF) capacity of lipophilic chains.

## Introduction

Microorganisms usually occur as aggregates of microcolonies. This clustering can be associated with substratum attachment and matrix encapsulation, which forms biofilm and is ubiquitous in nature. This mode of growth is essential to the survival of some microorganisms thriving in nutrient-depleted conditions and extreme environments (Hall-Stoodley et al., 2004). Because of this protective form, pathogenic bacteria like *Staphylococcus epidermidis* and *Pseudomonas aeruginosa* have become increasingly resistant to antibiotic treatments (Singh et al., 2021). The apparent medical implication of biofilm suggests a need for understanding its complex system to optimize possible mitigation strategies.

Biofilm formation usually starts with the attachment of free-living planktonic cells to a suitable substratum. Initial adherence of bacterial cells can be influenced by environmental factors such as pH, material type, temperature, and hydrophobicity (Dhar and Han, 2020). During this reversible stage, attachment is also mediated by microbial inherent properties like pili, flagellum, surface adhesins, and signaling molecules. This is followed by an irreversible stage of microbial network maturation with distinct Extracellular Polymeric Substance (EPS) formation. EPS is a mixture of protein, nucleic acids, and polysaccharides, which may vary per pathogen (Arciola et al., 2015). This encapsulation allows the bacteria to store nutrients, block drug penetration, and facilitate communication via quorum sensing. Ultimately, planktonic cell dispersal can happen on the outer layer of matured biofilms, which can spread and mediate systemic infections in clinical settings (Dhar and Han, 2020).

The marine ecosystem remains a rich repository of bioactive compounds (Jiménez, 2018; Petersen et al., 2020; Deng et al., 2022). Various host organisms and associated microbes were reported as good sources of antibiofilm compounds (Deng et al., 2022). Several marine-derived compounds were characterized to produce anti-biofilm properties with varying target domains, such as quorum sensing (QS)-inhibitor psammaplin A (Oluwabusola et al., 2022), EPS-disruptor cyclic dipeptide (L-Leucyl-L-propyl) (Gowrishankar et al., 2014), pre-formed biofilm suppressor butenolide (Yin et al., 2019), and bacterial aggregation downregulator rodriguesine A (Lima et al., 2014). Three modified fatty acids were previously purified from the shipworm endosymbiont *Teredinibacter turnerae* 991H.S.0a.06 (Lacerna et al., 2020). Turneroic acid, a new modified oxylipin characterized by an epoxide moiety and a hydroxy group, inhibited biofilm formation in *S. epidermidis* RP26A (Lacerna et al., 2020). Preliminary structure-activity relationship (SAR) showed that *trans*-12,13-epoxyoctadecanoic acid (**1**) has a high selectivity index for anti-biofilm activity against *S. epidermidis* with no antiproliferative effects against MDCK NBL-2 cell line (Lacerna et al., 2020). SAR showed the importance of the epoxide moiety in selective biofilm inhibition against *Staphylococci* (Lacerna et al., 2020).

Related compounds to **1** have been reported to exhibit different bioactivities but have not been assessed for antibiofilm properties in representative microbial pathogens. The saturated fatty acids palmitic (**2**) and stearic (**3**) acids are ineffective as antibiotics or biofilm inhibitors (Sanabria-Ríos et al., 2020). The racemic mixture of 9,10-epoxyoctadecanoic acid demonstrated antimicrobial activity against a *Pseudomonas syringae* strain but was ineffective against other subtypes and other plant pathogens including *Xanthomonas* and *Erwinia* (Prost et al., 2005). Meanwhile, 12,13-epoxy-9(*Z*)-octadecenoic acid inhibited the plant fungi *Phytophthora* and *Cladosporium* (Prost et al., 2005).

Herein, we report our efforts to expand the SAR using additional analogues of modified oxylipins and further elucidate its antibiofilm mechanism. We assessed the effects of modified oxylipins on the different stages of biofilm formation using static microdilution assays, microscopy, gene expression, and docking analysis.

## Materials and methods

### Compounds

**FIGURE 1:**
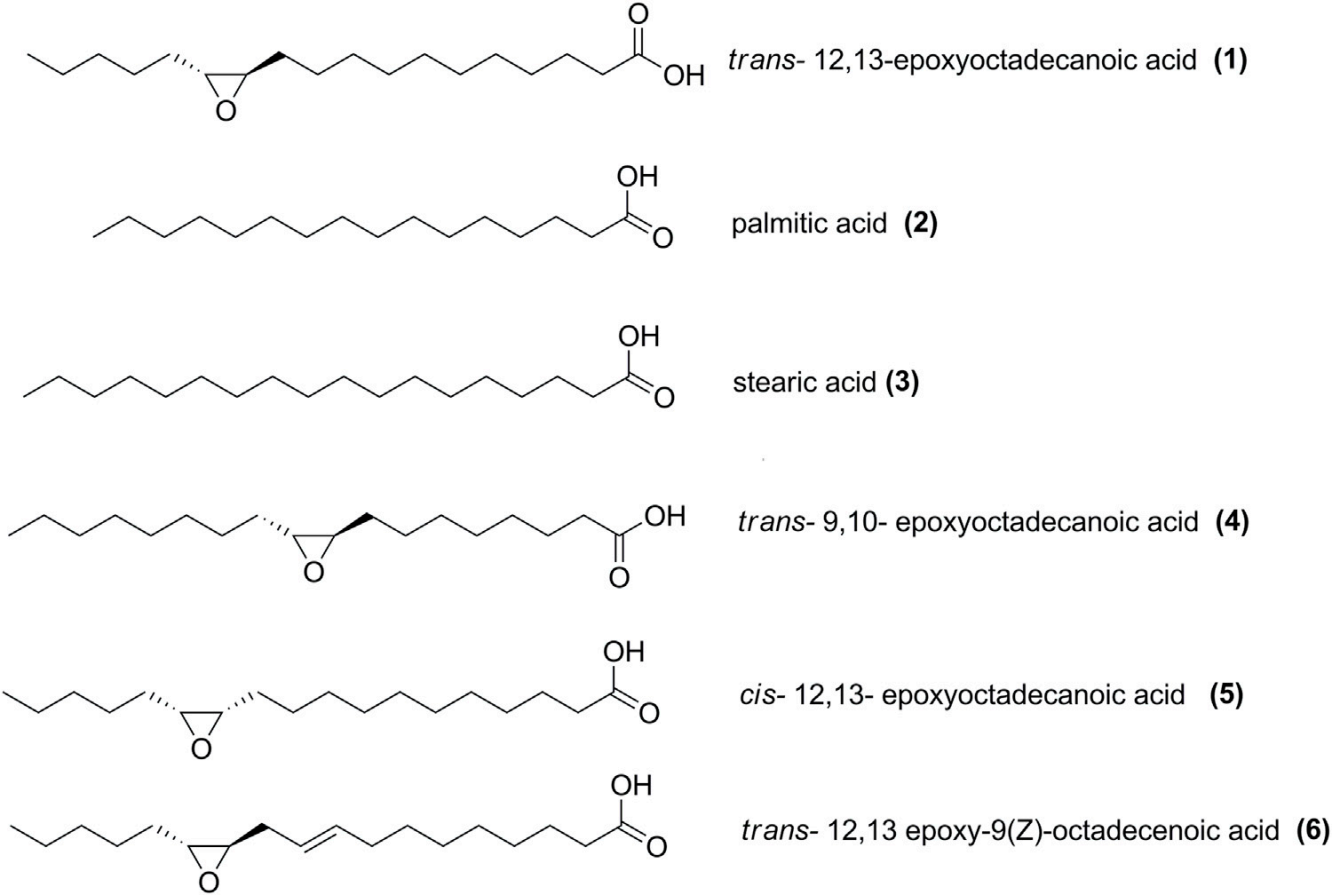
Structure of modified oxylipins used in this study.

Fatty acids palmitic (**2**) and stearic acids (**3**) were sourced from Sigma-Aldrich (St. Louis, MO, United States of America). *Trans*-12,13-epoxyoctadecanoic acid (**1**), *trans*-9,10-epoxyoctadecanoic acid (**4**), *cis*-12,13-epoxyoctadecanoic acid (**5**), *trans*-12,13-epoxy-9(*Z*)-octadecenoic acid (**6**) were purchased from Larodan Fine Chemicals (Solna, Sweden) (Figure 1). Stock solutions were prepared in DMSO. The positive control chloramphenicol was sourced from Sigma-Aldrich (St. Louis, MO, United States of America). Filtered sterilized DMSO (1%) was used as negative control.

### Bacterial cultures and culture conditions

Inoculum preparation was based on the methods of Lacerna et al. (2020), as adapted from Sarker et al. (2007). Briefly, Gram-(+) biofilm formers *Staphylococcus aureus* ATCC 6538 and *S.* *epidermidis* RP262A ATCC 35984 and Gram-(−) *P. aeruginosa* ATCC 41501 were revived on Tryptic Soy Agar TSA (Pronadisa, Spain) plates for biofilm studies and Mueller Hinton Agar (MHA) (Himedia, India) for planktonic setup. Tryptic Soy Broth (TSB) (Himedia, India) with 1% glucose and Mueller Hinton Broth (MHB) (Himedia, India) were used in the assays for biofilm and planktonic cells, respectively, unless otherwise stated. Microbial cell culture was adjusted to match 0.5 McFarland standard and was further diluted, equivalent to 5 × 10^5^ colony forming units/mL (cfu/mL). The 96-well polystyrene flat bottom plates (Costar 3596, United States of America) and 6-well plates (Costar 3506, United States of America) were covered with the corresponding sterile polystyrene lid and sealed with a parafilm to ensure a tight lid.

### Biofilm inhibitory concentration (MBIC) and minimum inhibitory concentration (MIC) determination

Assessment of the biofilm inhibitory activity of **1-6** was based on the method of Lacerna et al. (2020), as adapted from Skogman et al. (2012). Microbial pathogens were treated with two-fold serial dilution of **1**-**6**, starting with 128 μg/mL to 1 μg/mL. MHB and TSB culture media were used for planktonic and biofilm quantitation, respectively. The 96-well flat-bottom polystyrene plates (Costar 3,596, United States of America) were incubated for 18–20 h at 37 °C without shaking to promote biofilm formation. The formed biofilm was quantified using Alexa Fluor™ 488 (WGA 488, Thermo Fisher, United States of America) probe (0.05 μg/mL). The bound dye was solubilized with 33% acetic acid and quantified spectrophotometrically at 485 nm/420 nm excitation filters and 528 nm/520 nm emission filters. In another set of plates, the planktonic cells were monitored at the end of the incubation and quantified using 0.02% resazurin (Sigma, United States of America). The fluorescence was measured at 530 nm excitation and 590 nm emission using a microplate reader (Biotek Synergy HT, Winooski, United States of America). Percent inhibition was calculated relative to the solvent control. MBIC and MIC are the lowest concentration where ≥98% biofilm and ≥98% planktonic cells inhibition, respectively, were observed.

### Pre-formed biofilm disruption assay

*S. epidermidis* was incubated on TSB culture medium for 18–20 h at 37 °C without shaking to allow biofilm formation in a 96-well flat bottom polystyrene plate (Costar 3596, United States of America). The planktonic cells were removed and replaced with fresh medium and treated with two-fold dilution of **1** and **4**, starting from 128 μg/mL to 4 μg/mL. At the end of the 18 h incubation, the metabolic activity in the biofilm was quantified using 3-(4,5-dimethylthiazol-2-yl)-2,5-diphenyl tetrazolium bromide (MTT) (Sigma Aldrich). MTT quantifies viability through the conversion of the yellow tetrazolium MTT dye to a purple formazan product by mitochondrial reductases. The metabolic activity was assessed by reading the absorbance at 570 nm (Biotek Synergy HT, Winooski, United States of America). In a duplicate plate, the microbial density of the biofilm was assessed using WGA 488 probe according to the methods described above. Biofilm disruption was assessed relative to the solvent control (1% DMSO).

### Time-kill kinetics

A 24-h monitoring of *S. epidermidis* biofilm and planktonic growth was performed using TSB and MHB, respectively. Each setup in a 96-well polystyrene flat bottom plate (Costar 3596, United States of America) was treated with **1** and **4** (24 μg/mL) or solvent control (1% DMSO). Planktonic cell growth was assessed using MTT (Sigma Aldrich, United States of America) while biofilm was quantified using WGA 488 reagent. Absorbance and fluorescence readings were taken every 2 h using Biotek Synergy HT (Winooski, United States of America).

### Microscopy

Sterile glass coverslips (Thickness #1) were placed in 6-well flat bottom polystyrene plate (Costar 3506, United States of America). Briefly, *S. epidermidis* adjusted inoculum was transferred on each well, followed by TSB containing 24 μg/mL of compounds **1** or **4** (1% DMSO). Plates were incubated at 37°C for 24 h without shaking to facilitate biofilm formation.

The microbial content was aspirated at the end of incubation, and fixed with methacarn solution (methanol: chloroform: glacial acetic acid, 6: 3: 1) for 48 h (Dassanayake et al., 2020). Biofilms were rinsed with sterile Phosphate Buffer Saline (PBS), post-fixed with 1% osmium tetroxide (Electron Microscopy Science, Hatfield, United States of America) for 30 min, and washed with distilled water every 5 min (4x). Finally, the coverslips were dehydrated by an increasing series of ethanol: 30% for 5 min, 50% for 10 min, 70% for 15 min (2×) 95% for 20 min (2×), and 100% for 20 min (2×). Further chemical drying was done using increasing concentration of hexamethyldisilazane (HMDS) (Sigma Aldrich, Germany) every 20 min (1:1 EtOH:HMDS, 1:3 EtOH:HMDS, and 100% HMDS). Platinum coating for 40 s and 40 mAmps was conducted using Hitachi MC1000 sputter coater. SEM imaging was performed using a Hitachi SU Field Emission Scanning Electron Microscope.

### RNA extraction, quantitative real time- PCR analysis

*S. epidermidis* was cultured in TSB medium in a flat bottom polystyrene plate (Costar 3,506, United States of America). Compounds **1** and **4** (24 μg/mL), or 1% DMSO were added to plates and incubated at 37°C without shaking. At the end of the 24 h incubation, RNA extraction was performed on the total *S. epidermidis* population, consisting of both planktonic cells and biofilm, according to Juhlin et al. (2017) with some modifications. Bacterial lysis was performed using acid-washed glass beads (400–600 μm; Sigma Aldrich, United States of America) and TriZol-chloroform phase separation (Juhlin et al., 2017). Total RNA was extracted from the aqueous phase using Qiagen RNeasy^®^ Mini Kit according to the Manufacturer’s protocol. The purity and quantity of RNA were assessed using NanoSpec and Qubit™ RNA BR assay. RNA was further reverse transcribed into cDNA using SuperScript IV Reverse Transcriptase (Invitrogen, Lithuania). Gene expression was quantitatively determined by real-time PCR (Applied Biosystems™ ABI 7500) using SYBR Select Master Mix (Applied Biosystems, Thermo Fisher Scientific). The housekeeping gene *gyrB* was used to normalize the relative expression data of the genes of interest using the 2^−ΔΔCT^ method. Primer sequence of the target genes are as follows: *icaR* forward 5′-CATTGACGGACTTTACCAGTTTT-3′, and reverse 5′-ATCCAAAGCGATGTGCGTAG-3’; *icaB* forward 5′-GAAACAGGCTTATGGGACTTTG-3′, and reverse 5′-CAAGTGCGCGTTCATTTTT-3’; and *gyrB* forward 5′-TGACGAGGCATTAGCAGGTT-3′, and reverse 5′-GTGAAGACCGCCAGATACTTT-3’.

### Docking study

The structure of oxylipin **4** was obtained from PubChem (CID: 15,868) while the crystalline structures of Staphylococcal accessory regulator A (SarA) (PDB ID: 2FNP) and accessory gene regulator (AgrA) (PDB ID: 4XYO) were recovered from Protein Data Bank. Other ligands (**1** and **5**) were created using ChemDraw Ultra 12.0 (Cousins, 2005) and optimized using Avogadro (Hanwell et al., 2012). *In silico* molecular docking was carried out using AutoDockTools version 1.5.7 (Goodsell and Olson, 1990). Prior to docking, the water molecules from the protein were removed. Kollman charges were assigned while polar hydrogen bonds were included in the existing macromolecule. The ligand torsion was made rigid between carbons C8-C11 to maintain the *trans-*configuration. Grid map was optimized to include the surrounding residue with grid point spacing of 0.375 Å for AgrA and 0.558 Å for SarA. Lamarckian genetic algorithm was used to analyze the docking process with 50 runs and 300 population size. The complex with the least binding free energy and high clustering record was selected. Docking results were visualized by PyMOL Molecular Graphics System Version 1.2r3pre Schrödinger, LLC; Protein-Ligand Interaction Profiler (PLIP) (Adasme et al., 2021); and UCSF Chimera (Pettersen et al., 2004).

### Staphyloxanthin quantification

The bacterial suspension of *S. aureus* ATCC 6538 was incubated with **1** or **4** (24 μg/mL) for 24 h at 37°C without shaking in a 6-well flat bottom polystyrene plate (Costar 3506, United States of America). Cell pellets were collected by centrifugation at 10,000 rpm for 10 min, washed with sterile PBS and resuspended in 150 µL methanol for 30 min at 55°C. The absorbance of the extracted carotenoid was measured at 465 nm using microplate reader (Biotek Synergy HT, Winooski, United States of America). The production of *S. aureus* pigment was calculated relative to the solvent control.

## Results

### Structural configurations of the epoxide moiety can influence the biofilm inhibitory activity of modified oxylipins

Oxylipins **1, 4, 5** and **6** inhibited biofilm formation, with MBIC ranging from 24 to 96 μg/mL against *S. epidermidis*, and 64–128 μg/mL against *S. aureus* (Table 1). The most potent oxylipins **1** and **4** both have a *trans*-epoxide but differed on the position of the epoxide moiety. In contrast, the *cis*-epoxide bearing oxylipin **5** showed two-fold lower activity. This suggests that the configuration of the epoxide moiety, but not the position, may be critical for the biofilm inhibitory activity. Compound **6** had close to four-fold higher MIC among the epoxide-bearing oxylipins and may suggest the negative impact of the presence of an unsaturation in the aliphatic chain for bioactivity. The saturated fatty acids **2** and **3** were the least effective in inhibiting biofilm formation, corroborating the importance of the epoxide moiety for bioactivity. The inhibitory activity of the *trans*-epoxide bearing oxylipins showed selectivity to the Gram-(+) *Staphylococcus* species, with no inhibitory activity against the Gram-(−) *P. aeruginosa*. Because of the potent activity of **1** and **4**, these were used to further characterize the mechanism of biofilm inhibitory activity of modified oxylipins.

**TABLE 1:** Minimum biofilm inhibitory concentration (MBIC) and minimum inhibitory concentration (MIC) of **1−6** against *Staphylococcus epidermidis* RP62A ATCC 35984, *S. aureus* ATCC 6538, and *P. aeruginosa* ATCC 41501 using WGA 488 and resazurin-based microdilution assays.^a^

	MBIC and MIC (μg/mL)					
Compounds	*S. epidermidis* RP262A ATCC 35984		*S. aureus* ATCC 6538		*P. aeruginosa* ATCC 41501	
	Biofilm	Planktonic	Biofilm	Planktonic	Biofilm	Planktonic
**1**	24	>128	64	>128	>128	>128
**2**	>128	>128	>128	>128	>128	>128
**3**	>128	>128	>128	>128	>128	>128
**4**	24	>128	64	>128	>128	>128
**5**	48	>128	128	>128	>128	>128
**6**	96	>128	128	>128	>128	>128
Chloramphenicol	8	8	16	16	50	50

^a^ Data is the mean of two independent biological replicates with three technical replicates each. MBIC, and MIC, defined as the lowest concentration causing ≥98% biofilm and ≥98% planktonic cells inhibition, respectively.

**FIGURE 2:**
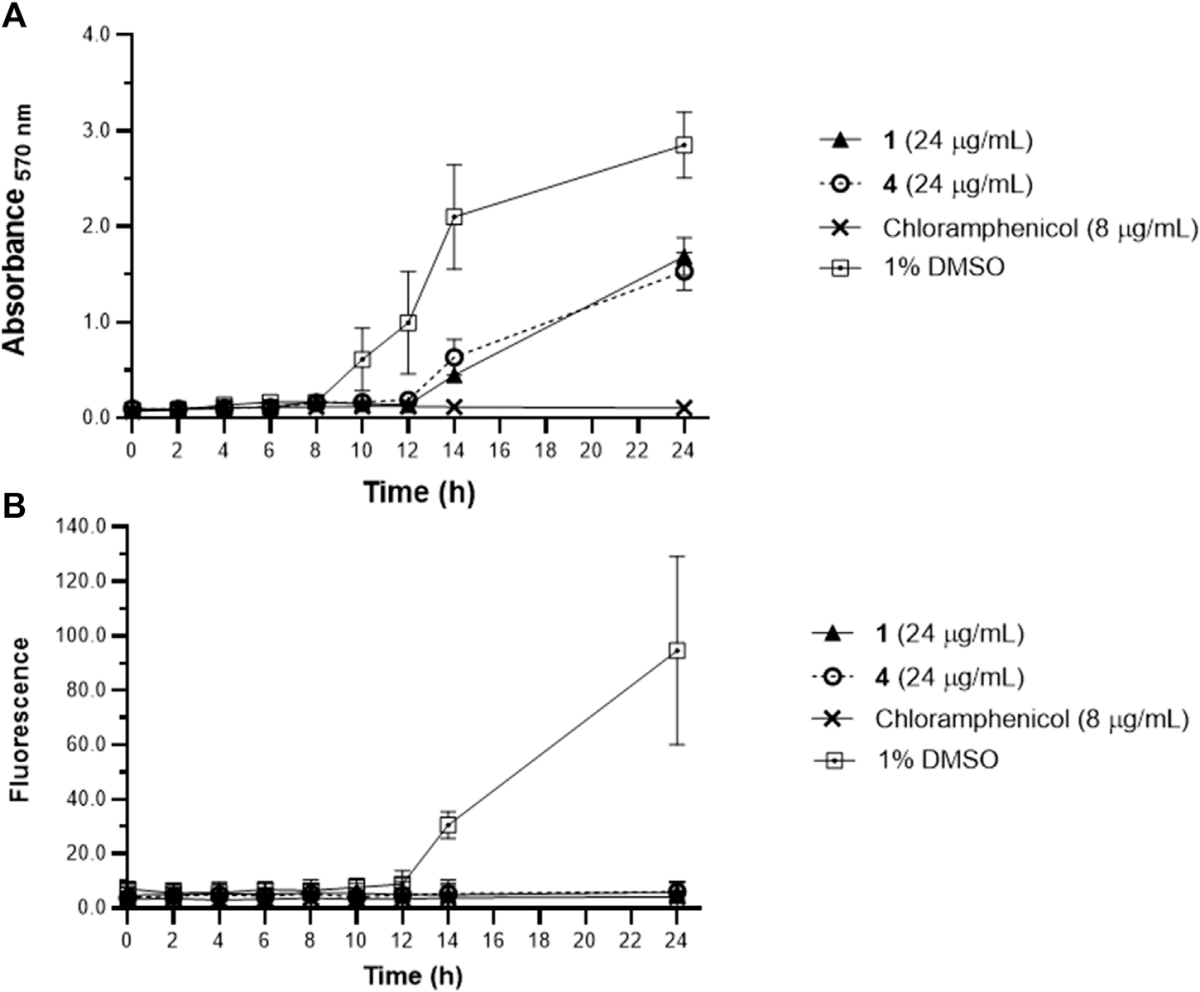
Metabolic activity of planktonic *Staphylococcus epidermidis* assessed by MTT **(A)**, and biofilm growth quantified by WGA 488 **(B)** from time-kill assay. *Staphylococcus epidermidis* were treated with **1** and **4** at MBIC = 24 μg/mL and the metabolic activity and biofilm were monitored every 2 h. Reduced metabolic activity was observed at 10 h post-incubation with **1** and **4**. Data are presented as mean ± SD of two independent biological replicates performed in triplicate (n = 6).

Further validation was done through a time course monitoring of the metabolic activity and biofilm formation in *S. epidermidis* for 24 h (Figure 2). Reduced metabolic activity was evident starting at 10 h post-incubation with **1** and **4** at 24 μg/mL. Biofilm matrix was absent in oxylipin-treated wells (Figure 2) and suggests selective targeting of **1** and **4** against microbial attachment of *S. epidermidis* during biofilm formation.

### Oxylipins can reduce metabolic activity but are unable to disperse mature biofilm

**FIGURE 3:**
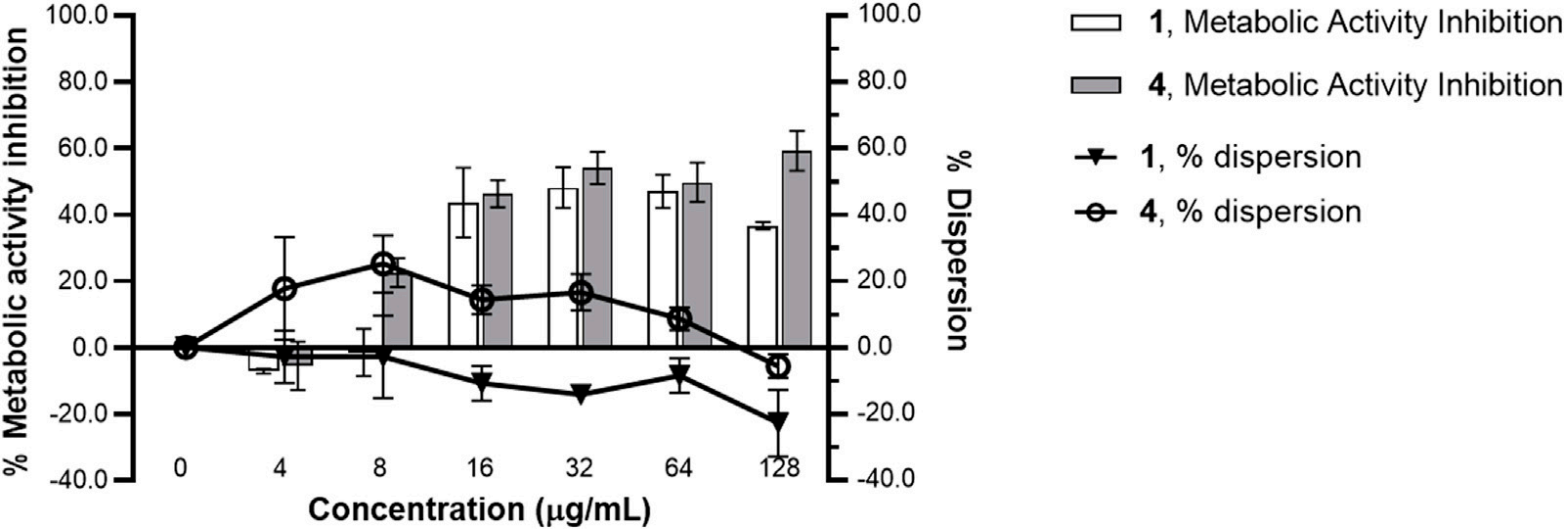
Metabolic inhibition and dispersion profile of increasing concentrations of **1** and **4** against *Staphylococcus epidermidis* RP62A ATCC 35984 pre-formed biofilm. A 24 h-old *Staphylococcus epidermidis* biofilm was treated with **1** and **4** from 0 to 128 μg/mL MBIC for 24 h. Using MTT reagent and WGA 488, metabolic activity and dispersive capacity, respectively, were measured. % metabolic activity and % dispersion was calculated relative to the solvent control (1% DMSO). A significant reduction in metabolic activity was observed with treatments of **1** and **4**. Data presented is representative of one biological replicate (mean ± SD) from two independent biological replicates with three technical replicates each.

Compounds **1** and **4** did not significantly disrupt mature *S. epidermidis* biofilm even at concentration up to 128 μg/mL (Figure 3). However, a concentration-dependent reduction in metabolic activity was observed in the pre-formed biofilm when treated with the oxylipins. A 36%–60% metabolic inhibition was observed at concentrations >8 μg/mL for both **1** and **4**.

### Oxylipins increased *icaR* gene expression

**FIGURE 4:**
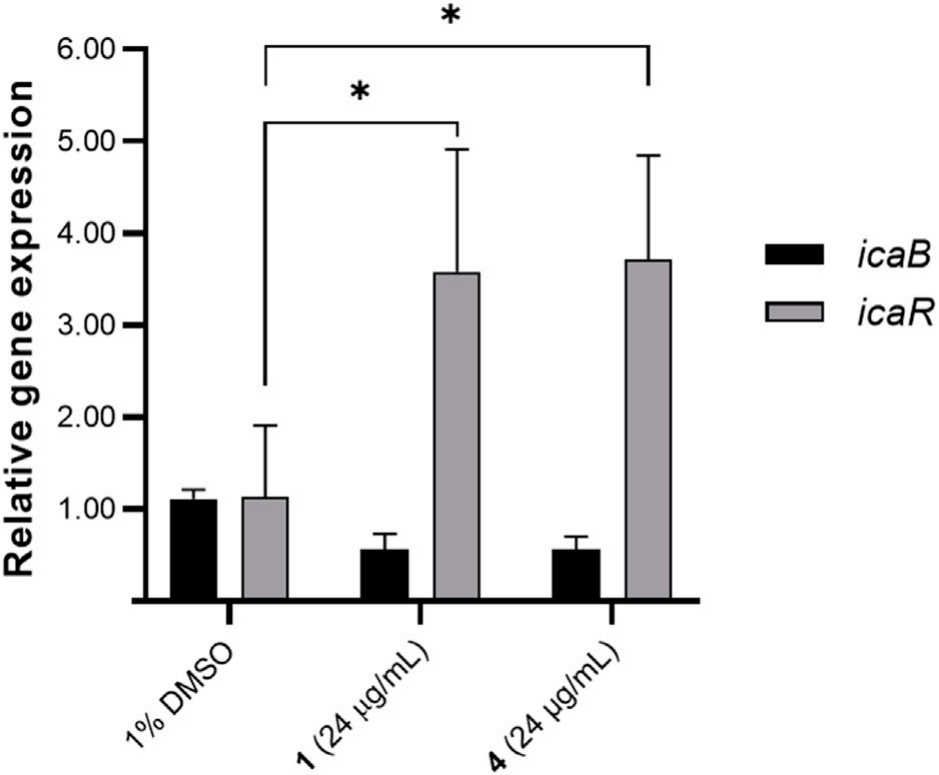
Effects of compounds **1** and **4** (24 μg/mL) on *icaB* and *icaR* expression. Data were analyzed using one-way ANOVA and Tukey’s *post hoc* test at *p*-value <0.05. Biofilm relevant genes *icaB* and *icaR* were differentially regulated by **1** and **4**. Data presented is representative of one biological replicate (mean + SD) from two independent biological replicates with four technical replicates.

To provide insights to the molecular mechanisms underlying biofilm formation, we assessed the effects of **1** and **4** on the *ica* operon. We prioritized the gene expression analysis for *icaR* and *icaB* given the clear association of these with biofilm formation. These two genes from the *icaADBC* transcription machinery are involved in extracellular matrix production and essential in biofilm attachment and structuring (François et al., 2023). *icaB* is responsible for the deacetylation and polymerization of *N*-acetylated β-1,6-linked *N*-acetylglucosamine (PNAG), while *icaR* encodes for the repressor of *ica* operon (François et al., 2023). The effect of **1** and **4** on the gene expression of biofilm-relevant genes *icaB* and *icaR* was determined using qRTPCR. A significant increase in *icaR* transcript level was observed (Figure 4). There was an ∼3.58- and 3.71-fold increase in *icaR* expression with 24 μg/mL treatment of **1** and **4**, respectively. Consequently, *icaB* expression was downregulated by **1** and **4**, with 0.57 and 0.56-fold reduction in transcript level, respectively.

### Oxylipins decreased EPS and adherent micro-colonies

**FIGURE 5:**
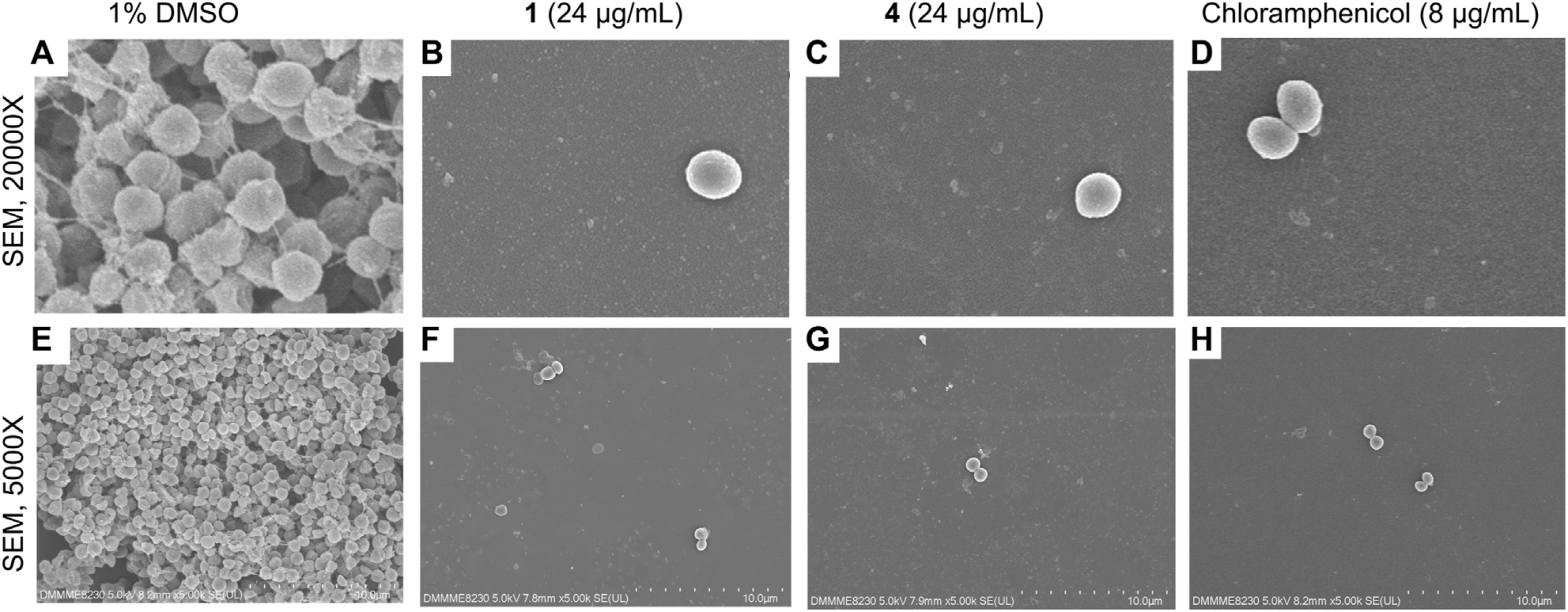
SEM micrographs of *Staphylococcus epidermidis* at ×20,000 magnification **(A–D)** and 5,000x **(E–H)** at 24-h post-incubation with **1** and **4** (24 μg/mL), positive control chloramphenicol (8 μg/mL), and solvent control (1% DMSO). Micrographs reveal reduction in attached cells and visible EPS with **1** and **4**.

SEM was performed to assess the phenotypic effects of the oxylipins on *S. epidermidis* morphology and biofilm. Untreated *S. epidermidis* showed the typical morphology, with circular microcolonies that are clumped together (Figure 5). Attachment of the cells is facilitated by EPS that appeared as thread-like structures in the SEM micrographs (Figure 5). Treatment with oxylipins **1** and **4** showed a significantly reduced number of attached cells and EPS (Figure 5). The observed phenotype with **1** and **4** treatments were significantly different from the negative control.

### Oxylipins putatively interact with quorum sensing regulator proteins

To substantiate the observed *ica* regulation and SEM observation, we assessed the interaction of **1, 4,** and **5** on the global regulatory protein SarA, which positively enhances *ica* operon and consequently, PIA/PNAG synthesis. SarA modulates key virulence factors which allows the transition from planktonic growth to biofilm state (Tormo et al., 2005). SarA interplays with the quorum sensing accessory gene regulator (AgrA) (Reyes et al., 2011). Given that SarA influences the *agr* pathway, we included AgrA to the *in silico* assessment. Additionally, oxylipins share structural similarity with known diffusible signal factors (DSFs) which also interferes with quorum sensing through the SarA pathway (Schmidt et al., 2003; Qazi et al., 2006; Biswas and Götz, 2022). We hypothesize that because of the structural similarity of these oxylipins with diffusible signal factors, compounds **1** and **4** are likely to have an analogous mechanism of action in inhibiting biofilm formation. Therefore, we used molecular docking to demonstrate the possible interactions of the modified oxylipins with Agr and SarA.

**FIGURE 6:**
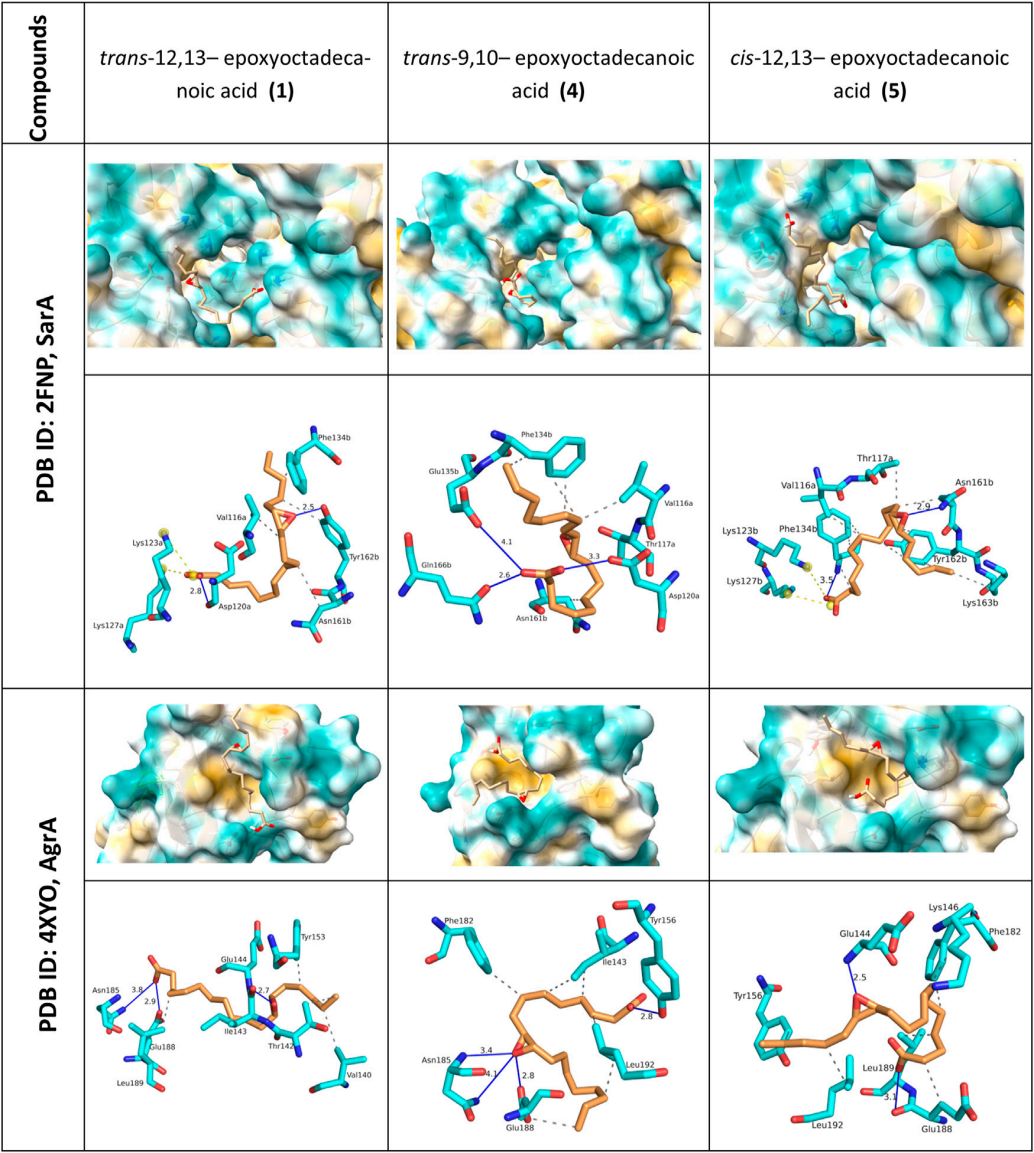
Visualization of molecular docking analysis of oxylipin analogues with AgrA and SarA. 3D surface interactions for both protein complexes are shown with closeup view in the protein surfaces. 2D interaction with AgrA and SarA are represented by hydrogen bonds D-A (blue lines), hydrophobic contacts (grey dashed line), and salt bridges (yellow dashed line). Critical interactions were observed between SarA and AgrA with the epoxide moiety of the oxylipins.

*In silico* docking of **1**, **4,** and **5** (Figure 6; Supplementary Table S1) was done using AutoDock (version 1.5.7). The oxylipin analogues were docked in helices α1 - α5 of SarA receptor. Estimated binding energy obtained from SarA ranged from −3.65, −5.03 and −3.79 kcal/mol from oxylipins **1**, **4**, and **5**, respectively (Supplementary Table S1). Hydrogen bonds were noted in the oxylipin-receptor complexes, with critical interactions formed with the epoxide and terminal carboxylic acid moiety (Figure 6; Supplementary Table S1). Hydrophobic interactions were seen in Val116a, Phe134b, Asn161b, Tyr162b.

In the case of AgrA, the binding energy obtained from the molecular docking ranged from −3.28, −4.85 and −2.76 kcal/mol from oxylipins **1**, **4**, and **5**, respectively. The compounds occupied a conserved hydrophobic site formed by helix α1-sheet β1 (Figure 6; Supplementary Table S1). Hydrogen bonding interactions of **1**, **4**, **5** with AgrA were largely similar, with key interactions observed with Asn185, Glu188, and Glu144.

### Oxylipins reduce carotenoid production in *S. aureus*

**FIGURE 7:**
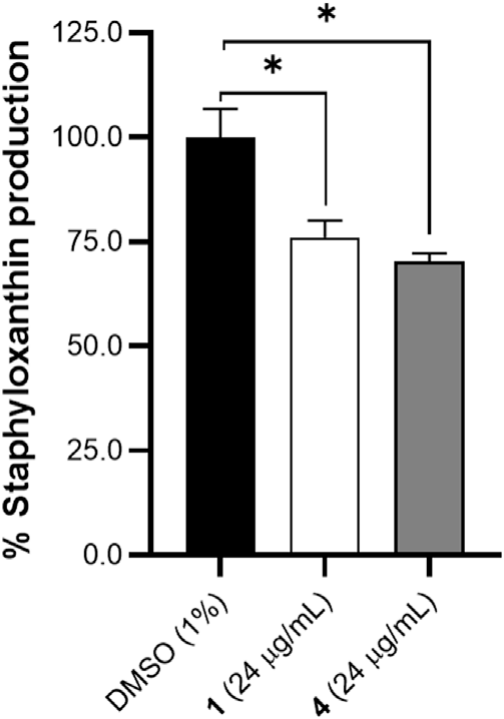
Effects of compounds **1** and **4** (24 μg/mL) on staphyloxanthin production in *S. aureus* after 24 h of treatment. A significant decrease in pigment was observed with treatment of **1** and **4**. Data were analyzed using one-way ANOVA with multiple comparisons at *p* < 0.05. Data is representative of one biological replicate (mean +SD (n = 3) from two independent experiments with three technical replicates each.

AgrA expression can influence several virulence factors including pigment formation in *S. aureus* (Tan et al., 2022). Both *S. aureus* and *Staphylococcus lugdunensis* with mutant *agrA* operon were found to have reduced pigmentation as compared to wildtype strains (Aubourg et al., 2022; Tan et al., 2022; Cella et al., 2023). On this basis, we validated the docking analysis of AgrA using staphyloxanthin quantification. Staphyloxanthin is quantified by carotenoid or yellow pigment production in *S. aureus*. Oxylipins **1** and **4** caused a 24% and 29% decrease in staphyloxanthin production, respectively (Figure 7). The decrease in staphyloxanthin production, and decreased EPS and microcolonies observed in the SEM corroborates the cellular permeability action of the oxylipins at the early stages of biofilm formation.

## Discussion

We further probe the mechanism of action and identify the structural determinants for the biofilm inhibitory activity of modified oxylipins. Our results corroborate the findings of Lacerna et al. (2020) and highlight the additional structural motifs that are responsible for the biofilm inhibitory activity of modified oxylipins. The addition of an unsaturation to the aliphatic chain negatively impacts the antibiofilm activity of this class of compounds. Positional isomers of modified oxylipins showed comparable bioactivity. This is analogous to the observation in cyclopropyl-bearing fatty acid, where similar inhibitory activities in *Escherichia coli* and *Pseudoroseovarius crassostreae* were obtained despite the modification involving the cyclopropyl unit (Amiri Moghaddam et al., 2018). The *trans*-epoxide **1** and **4** may provide an optimum spatial and electrostatic interaction with the cellular target, compared to its *cis*-isomer. SAR evaluation of cerulenin analogues revealed that *trans-*2,3-epoxydodecanoic acid is 5x more potent HIV protease inhibitor than *cis*-epoxydecenoic (Blumenstein et al., 1989). By disruption of peptidoglycan synthesis and fatty acid synthesis, increased antimicrobial activity was also reported from unsaturated linoleic and arachidonic acid as compared to their saturated counterpart (Zheng et al., 2005; Kenny et al., 2009; Casillas-Vargas et al., 2021).

Interestingly, these oxylipins are structurally related to the *P. aeruginosa* derived fatty acids (10*S*)-hydroxy-(8*E*)-octadecenoic acid (10-HOME) and 7*S*,10*S*-dihydroxy-(8*E*)-octadecenoic acid (7,10-DiHOME). 10-HOME and 7,10-DiHOME are upregulated during biofilm formation and virulence in *P. aeruginosa* (Martínez and Campos-Gómez, 2016; Martínez et al., 2019). In contrast to 10-HOME and 7,10-DiHOME, **1**-**6** did not affect biofilm formation in *P. aeruginosa*, whether inhibition or promotion. The epoxide-bearing oxylipins showed selective bioactivity against the Gram-(+) *S. aureus* and *S. epidermidis* and may suggest the role of the epoxide moiety not just for potent bioactivity but also for selectivity.

To further probe the cellular effects of these modified fatty acids, we undertook a gene to phenotype approach. We focused on *icaB* and *icaR* transcripts that are part of the *ica* operon responsible for the synthesis of polysaccharide intercellular adhesin/polymeric *N*-acetyl-glucosamine (PIA/PNAG) exopolysaccharides. *icaR* serves as a transcriptional repressor and regulates the activation of downstream *icaADBC* (François et al., 2023). *icaB* deacetylates and polymerizes PIA/PNAG in *S. aureus* and *S. epidermidis* resulting in enhanced protective layer and ideal surface adherence of PIA/PNAG (Cerca et al., 2007; Arciola et al., 2015). Significant upregulation of the *icaR* repressor can compromise the synthesis of EPS component polysaccharide intercellular adhesin (PIA) or polymeric *N*-acetyl-glucosamine (PNAG) under an *ica*-dependent pathway. Microbial isolates bearing single nucleotide polymorphisms in *icaR* showed decreased exopolysaccharide production (Morales-Laverde et al., 2022), directly demonstrating the impact of *icaR* on PIA/PNAG. Disruption of PIA/PNAG can impair not just the structural integrity of the biofilm matrix but can also decrease the pathogenicity and virulence of *Staphylococcus* (Nguyen et al., 2020; Cheung et al., 2021; Peng et al., 2023). Treatment of **1** and **4** showed an increase in *icaR* transcript level that consequently translated to a modest decrease in *icaB*. SEM analysis confirmed a decline in EPS in oxylipin-treated *S. epidermidis* compared to the solvent control. The observed cellular effects of **1** and **4** are similar to those observed for quercetin, gallic acid and (+)-nootkatone, where suppression of biofilm formation is obtained through modulation of the *ica* operon in Staphylococci (Guo et al., 2021; Mu et al., 2021).

The *ica* operon is regulated by the Staphylococcal accessory regulator (SarA) and consequently, by the quorum sensing accessory gene regulator (AgrA). SarA is a DNA binding protein that belongs to the global modulators of various virulence factors in Staphylococci and increases the production of toxins, fibronectin, and fibrinogen (Cheung et al., 2008). SarA can regulate the *ica* operon and the quorum sensing regulator *agr* operon (Peng et al., 2023). SarA binds to the *ica* operon with high affinity and enhances *ica* expression (Tormo et al., 2005). Interaction of SarA with AgrA leads to enhancement of the conformational bending of promoter during *agr* locus transcription (Reyes et al., 2011).The *ica* operon is, however, not affected directly by *agr* expression. The *agr* pathway is often associated with virulence, pathogenesis, and toxin production in *S. aureus* (Le and Otto, 2015). The *agr* operon is involved in both biofilm formation and dispersion during the maturation stage (Le and Otto, 2015). A functional *agr* pathway is important in biofilm structural integrity but in some cases can induce biofilm formation (Gray et al., 2013; Le and Otto, 2015). Despite the divergent phenotypic expressions of *agr* QS system, several authors still consider the *agr* system to be prospective targets in biofilm mitigation studies (Park et al., 2007; Gray et al., 2013; Ganesh et al., 2022). Specific interference with AgrA-DNA binding domain is one of the possible ways to decrease or block the RNA III concentration (Gray et al., 2013).

Several diffusible signal factors (DSF) composed of short to medium chain alkyl chains were reported to regulate cell communication in prokaryotes (Kumar et al., 2020). Hydroxy-bearing DSF such as the 3-hydroxyhexadecanoic acid from *Ralstonia solanacearum* (Flavier et al., 1997), is structurally similar to the oxylipins tested. Due to limited protein structures in *S. epidermidis*, we used the crystallized structures of staphylococcal accessory regulator (SarA) and accessory gene regulator A (AgrA) obtained from closely related *S. aureus*. Both AgrA and SarA proteins in *S. aureus* and *S. epidermidis* displayed sequence homology ranging from 68% to 84% (Fluckiger et al., 1998; Otto et al., 1998; Van Wamel et al., 1998).

At the molecular level, our docking assessment showed that oxylipins occupy a hydrophobic pocket in both SarA and AgrA, with structural changes possibly resulting in alteration of specific amino acid binding sites. While the binding pockets occupied by **1** and **4** in SarA and AgrA are not directly involved in target gene binding, this may possibly disrupt the stability of the interaction with the consensus target gene. The observed activity of the compounds may be attributed to the steric hindrance introduced while interacting with multiple amino acids involved in enhancing the stability with the consensus target gene. Leu186 and Lys187 are critical amino acids in AgrA that form hydrogen bonding interactions with the promoter DNA, leading to stabilization of the complex (Rajasree et al., 2016). In comparison with other epoxide bearing QS inhibitors such as cerulenin, fosfomycin, and ambuic acid, **1** and **5** also showed similar hydrogen bonding interactions with Glu144 and Lys146 in the AgrA complex (Refai et al., 2023). The binding of **1**, **4**, and **5** to SarA is reminiscent of the interactions of other natural products carvacrol, eugenol, and hesperidin (Selvaraj et al., 2020; Vijavyakumar et al., 2022). Key interactions were observed in Asn161b, Thr117a, Gln166b (Selvaraj et al., 2020; Vijayakumar et al., 2022).

The molecular docking results were validated by the differential gene regulation of the *icaR* and *icaB* and staphyloxanthin levels. RNA III-independent *agr* pathway is involved in carotenoid production in *S. aureus* (Queck et al., 2008). Mutations on *agr* can lead to variations in virulence factors including loss of pigment production, hemolysin activity, and oxidative stress regulation (Aubourg et al., 2022; Tan et al., 2022). The impact of *agr* gene expression levels on biofilm formation is, however, less definitive compared to other genes such as *icaA* and *icaR* (Arciola et al., 2015). Phenotypic variations in virulence factors have been observed in *agr*-related studies, a case from myriad of factors including staphylococcal strain dependency, compound dosage response, and culture conditions (Yarwood and Schlievert, 2003; Pant et al., 2022; 2023). The molecular interactions and phenotypic observation is congruent with the observations for 3-hydroxybenzoic acid (Ganesh et al., 2022) and ω-hydroxyemodin (Daly et al., 2015) as disruptors of biofilm formation in *S. aureus*.

Staphyloxanthin production in Staphylococci is relevant not just in aggregation during biofilm formation, but also contribute to other virulence factors such as regulating toxic free radicals, and recognition of neutrophil attack (Elmesseri et al., 2022; Cella et al., 2023). A decreased staphyloxanthin production was observed for treatment with compounds **1** and **4**. Staphyloxanthin production is an important adaptation for *S. aureus* by acting as a scavenger of reactive oxygen species (Elmesseri et al., 2022). Inhibition of this phenotype clearly demonstrates the cellular permeability of the modified oxylipins in *S. aureus*. At the same time, the SEM experiment using *S. epidermidis* showed very distinct growth morphology with solvent control, further demonstrating the absorption of compounds **1** and **4**.

## Conclusion

Effective mitigation strategies against biofilm formation are crucial for public health. The ability of *S. epidermidis* to colonize biomaterials and to cause persistent clinical infections remains a threat worldwide. The *trans*-epoxide moiety in *trans*-9,10-epoxyoctadecanoic acid (**1**) and *trans*-12,13- epoxyoctadecanoic acid (**4**) imparts selectivity and potent activity against biofilm formation in *S. epidermidis.* The *trans*-epoxide bearing modified oxylipins modulate the transcript levels of key biofilm target genes and consequently, decreasing the phenotypic traits of biofilm formation such as EPS and pigment production.

## Data Availability

The data presented in the study are deposited in FigShare, http://doi.org/10.6084/m9.figshare.25745811.
