# Supplementary material for: Modified oxylipins as inhibitors of biofilm formation in Staphylococcus epidermidis
Source: Front Pharmacol. 2024 May 23;15:1379643. doi: 10.3389/fphar.2024.1379643 (PMC11153713; doi:10.3389/fphar.2024.1379643)
Supplement: Supplementary file 1 [file DataSheet1.PDF]

Modified oxylipins as inhibitors of biofilm formation in *Staphylococcus epidermidis*

Jacquelyn E. Peran, Lilibeth A. Salvador-Reyes

Marine Science Institute, College of Science, University of the Philippines Diliman, Velasquez St., University of the Philippines Diliman, Quezon City 1101, Philippines

*** Correspondence:** lsreyes@msi.upd.edu.ph

1 Supplementary Figures and Tables

Supplementary Table 1. Summary of molecular interactions between *S. aureus* SarA and AgrA with different oxylipin analogues.

PDB ID	Ligand	Estimated binding energy (kcal/mol)	Hydrogen Bonding	H-bond Distance (Å) D-A	Hydrophobic Contacts	Hydrophobic interaction Distance (Å)	Salt Bridges
2FNP: SarA	1	-3.65	Asp120a,<br>Tyr162b	2.8<br>2.5	Val116a,<br>Phe134b,<br>Asn161b,<br>Tyr162b	3.4<br>3.2<br>3.9<br>3.8	Lys123a,<br>Lys 127a
	4	-5.03	Asp120a<br>Glu135b,<br>Gln166b	3.3<br>4.1<br>2.6	Val116a,<br>Thr117a,<br>Phe134b,<br>Asn161b	3.2<br>3.4<br>3.2, 3.6,<br>3.6	NA
	5	-3.79	Phe134b,<br>Asn161b	3.5<br>2.9	Val116a,<br>Thr117a,<br>Phe134b,<br>Asn161b,<br>Tyr162b,<br>Lys163b	3.3<br>3.5<br>3.3, 3.5<br>3.6<br>3.9, 3.4, 3.7<br>3.7	Lys123b,<br>Lys127b
4XYO: AgrA	1	-3.28	Glu144<br>Asn185<br>Glu188	2.7<br>3.8<br>2.9	Val140,<br>Thr142,<br>Ile143,<br>Tyr153,<br>Leu189	3.7<br>3.9<br>3.3<br>3.3<br>3.6	NA
	4	-4.85	Ty156,<br>Asn185,<br>Asn185,<br>Glu188	2.8<br>3.4<br>4.1<br>2.8	Ile143,<br>Phe182,<br>Glu188,<br>Leu192	3.6 and 3.1<br>3.4<br>3.8<br>3.2	NA
	5	-2.7	Glu144,<br>Glu188	2.5<br>3.1	Lys146,<br>Tyr156,<br>Phe182,<br>Glu188,<br>Leu189,<br>Leu192	3.5<br>3.9<br>3.6<br>3.7<br>2.9<br>3.6	NA
